# Cicada Wing Inspired Template-Stripped SERS Active 3D Metallic Nanostructures for the Detection of Toxic Substances

**DOI:** 10.3390/s21051699

**Published:** 2021-03-02

**Authors:** Srijit Nair, Juan Gomez-Cruz, Gabriel Ascanio, Aristides Docoslis, Ribal Georges Sabat, Carlos Escobedo

**Affiliations:** 1 Department of Chemical Engineering, Queen’s University, Kingston, ON K7L 3N6, Canada; srijit.nair@queensu.ca (S.N.); j.gomezcruz@queensu.ca (J.G.-C.); docoslis@queensu.ca (A.D.); 2 Instituto de Ciencias Aplicadas y Desarrollo Tecnológico (ICAT), Universidad Nacional Autónoma de México (UNAM), Cto. Exterior S/N, C.U., Coyoacán, Ciudad de México 04510, Mexico; gabriel.ascanio@icat.unam.mx; 3 Department of Physics and Space Science, Royal Military College of Canada, Kingston, ON K7K 7B4, Canada; sabat@rmc.ca

**Keywords:** bioinspired nanostructure, surface-enhanced Raman spectroscopy, surface relief gratings, melamine detection, cicada wing nanostructure

## Abstract

This article introduces a bioinspired, cicada wing-like surface-enhanced Raman scattering (SERS) substrate based on template-stripped crossed surface relief grating (TS-CSRG). The substrate is polarization-independent, has tunable nanofeatures and can be fabricated in a cleanroom-free environment via holographic exposure followed by template-stripping using a UV-curable resin. The bioinspired nanostructures in the substrate are strategically designed to minimize the reflection of light for wavelengths shorter than their periodicity, promoting enhanced plasmonic regions for the Raman excitation wavelength at 632.8 nm over a large area. The grating pitch that enables an effective SERS signal is studied using Rhodamine 6G, with enhancement factors of the order of 1 × 10^4^. Water contact angle measurements reveal that the TS-CSRGs are equally hydrophobic to cicada wings, providing them with potential self-cleaning and bactericidal properties. Finite-difference time-domain simulations are used to validate the nanofabrication parameters and to further confirm the polarization-independent electromagnetic field enhancement of the nanostructures. As a real-world application, label-free detection of melamine up to 1 ppm, the maximum concentration of the contaminant in food permitted by the World Health Organization, is demonstrated. The new bioinspired functional TS-CSRG SERS substrate holds great potential as a large-area, label-free SERS-active substrate for medical and biochemical sensing applications.

## 1. Introduction

Biomimicry is an emerging field with the objective of replicating physical or chemical attributes found in nature to create human-made devices. The development of biomimetic materials and devices has been particularly useful in optics and sensing applications [1,2]. Examples of bioinspired materials include polymer-based biohybrid sensor interfaces [3], functional nanostructures of S-proteins for breast cancer cell detection [4], wearable eye health monitoring sensors [5], anti-Moiré grids with the optoelectronic performance [6] and SERS substrates inspired by the geometry of lotus seedpod [7]. Subwavelength periodic structures, such as nipple arrays and tapered pillars, can be found in some insect eyes and wings. Cicadas, in particular, have tapered nanopillars in their transparent wings to suppress light reflection, which makes them invisible to predators [8] and provides them with self-cleaning, superhydrophobic and bactericidal properties [9]. The amplitude and periodicity of the nanopillars range between 170 and 300 nm in order to achieve minimal reflection in the visible 300–800 nm spectrum [10,11,12,13]. In previous studies, direct deposition of metals on cicada wings has been used to investigate broadband light absorption properties of the nanostructures [14], as surface-enhanced Raman scattering (SERS) substrates [15,16] and to produce bio-templated SERS-active nanostructures transferred to optical fibers [17]. Other studies include photocatalytically deposited metallic nanoparticles on cicada and butterfly wings [18]. However, these methodologies enable the production of fixed-pitch nanostructures, preventing the tailored fabrication of SERS active surfaces of similar or identical morphologies.

Nanostructures that support surface plasmon resonance (SPR) have been widely used for sensing and biosensing applications through the use of different techniques, including SPR spectroscopy [19,20,21,22,23,24,25], SPR imaging [26,27,28,29] and surface-enhanced Raman scattering (SERS) spectroscopy [30,31,32,33,34,35,36,37]. SERS, particularly, allows for highly sensitive detection and specific identification of analytes. Nevertheless, metallic nanostructures must enable high enhancement of near-surface electromagnetic field intensities to qualify as SERS substrate [36]. Nano-engineered substrates such as metallic tips [35], nanohole arrays [38] and nanogratings [32] have been investigated for better controlled and reproducible SERS substrates. These structures provide a uniform enhancement over a large surface area, negating the concept of plasmonic “hot-spots” where only specific regions experience electromagnetic field strength enhancement [15]. Metallic nanogratings, in particular, experience large-area uniform electromagnetic enhancement, which increases the chances for analyte detection via SERS spectroscopy [39,40,41]. However, excitation of plasmons on 1D nanogratings is maximized when the polarization of the incident light is aligned with the grating vector [34]. Optimal enhancement is determined by the morphology of the nanogratings and the relative angle between the incoming light polarization and the grating vector. The polarization dependency of the nanostructures can be overcome by structuring them into a 2-dimensional (2D) arrangement. Crossed relief gratings (CSRGs) are 2D nanostructures that enable polarization-independent SERS detection, offering enormous potential for specific analyte sensing [19,21,26]. SPR excitation by one of the superimposed gratings is re-radiated by its orthogonal counterpart in a polarization state that is orthogonal to that of the incident light. Metallic CSRG may enhance the electromagnetic field intensity at a metal-dielectric boundary near-surface region by ~30 times, but they are fabricated from an azobenzene molecular glass (gDR1) solution that consists of azobenzene chromophore molecules, which are SERS active. This aspect has limited the deployment of CSRGs for SERS-based analysis as target signals may get masked by the azobenzene Raman spectra. One way to tackle this problem, and the main motivation of this work, is by replicating the tapered nanopillars in the cicada wings, to take advantage of their optical properties, and to use template stripping to allow the transfer of the metallic nanostructures to another substrate without the gDR1 layer that could potentially mask SERS signals, with the additional benefit of being pitch-customizable to provide antireflective (AR) or signal generation properties at desired wavelengths. Template stripping is a cost-effective and cleanroom-free approach that has been used for transferring other types of metallic nanostructures while preserving their shape and plasmonic efficiency [42,43].

Here, we present a polarization-independent, template-stripped Ag CSRG (TS-CSRG) SERS substrate, inspired by the tapered nanopillars found in the Cicada wings [15], along with the outstanding plasmonic capabilities of subwavelength metallic CSRG. The new methodology is achieved using holographic exposure and template-stripping of silver-coated CSRG using a UV-curable epoxy that enables fabrication of homogeneous, pitch-customizable, large-area, and low-cost substrates that allows for reproducible SERS signals. FDTD simulations are used in the design process to confirm the enhancement and distribution of the electromagnetic field along the nanostructures. The pitch-dependency of the TS-CSRG is used to tailor the SERS signals response upon the adsorption of Raman reporter molecule Rhodamine 6G (R6G). To showcase the capabilities of Ag TS-CSRG as SERS substrates in a real sensing context, we demonstrate the effective, label-free detection of melamine at concentrations of 1 ppm, which corresponds to the maximum residue limit for melamine in infant formula dictated by the World Health Organization (WHO) [44].

## 2. Materials and Methods

### 2.1. Atomic Force Microscopy

Imaging of the cicada wings, CSRGs, and TS-CSRGs structures was performed using a Dimension Edge atomic force microscope (AFM) system (Bruker, Billerica, MA, USA). A ScanAsyst-Air AFM tip (Bruker, Billerica, MA, USA) was utilized to scan a 5 µm × 5 µm area, using the peak-force tapping mode, with a scan rate of 1 Hz per line. Bruker NanoScope Analysis software was used to fit and analyze the AFM scans and obtain parameters such as the topography, depth, and pitch of the structures.

### 2.2. Fabrication of Nanogratings

The surface plasmon resonance wavelength of CSRGs is found by matching the SPR wavevector to the diffracted light via the grating equation so that the following equality is obtained [19]:

*k_sp_* = *k*_0_*nsinθ* ± 2*πm*/*Λ*, (1)

where *k_sp_* is the surface plasmon wave number, *k*_0_ is the incident light wave number in free space, *n* is the refraction index of the dielectric, *θ* is the incidence angle, *m* is the diffraction order (normally limited to unity), and *Λ* is the grating pitch.

Fabrication of the CSRGs was performed using the rapid and high-throughput interferometric technique described elsewhere [19,21,26]. Azobenzene molecular glass (gDR1) solution (DR1-glass, 2.99 mM, 94%) was prepared according to the methods described elsewhere [45]. A volume of 500 µL of 3 wt % gDR1 solution, diluted in dichloromethane, was spin-coated on a 2.5 cm × 2.5 cm Corning 0215 soda lime microscope glass slide (Ted Pella Inc., Redding, CA, USA) using a Headway Research spin-coater (Headway Research Inc., Garland, TX, USA) at 1000 RPM for 20 s. The spin-coated samples were then dried and annealed for 1 h at 90 °C in a Yamato ADP-21 oven (Santa Clara, CA, USA) to generate a uniform gDR1 film of approximately 200 nm thick, verified by a Sloan Dektak II surface profiler (Veeco Instruments Inc., Plainview, NY, USA). CSRGs were written on the gDR1-coated substrates by direct holographic exposure to the laser-light interference pattern assisted by a Lloyd mirror optical setup. The laser beam from a solid-state diode-pumped laser (Coherent, Santa Clara, CA, USA, Verdi V6, λ = 532 nm, irradiance = 140 mW/cm^2^) was directed onto a Lloyd mirror optical setup to allow for molecular mass transport of the azo-molecules to generate of nanopatterned SRGs. After the initial inscription of the SRGs (time of exposure = 300 s), the sample was rotated by 90° and a second exposure for 100 s was performed to fabricate orthogonally superimposed SRGs. An 80-nm layer of silver was subsequently sputtered over the CSRG using a Bal-Tec SCD 050 sputter-coater, to make an Ag-CSRG. The prepared samples had a periodicity of 450, 500, 550, 600 nm.

### 2.3. Template-Stripping Procedure

The fabricated Ag-CSRG was spin-coated with a UV-curable epoxy (NOA61, Norland Products Inc., East Windsor, NJ, USA) to generate uniform epoxy coating. Next, a pre-cleaned Corning 0215 soda lime microscope glass slide (Ted Pella Inc., Redding, CA, USA) was pressed against the epoxy-coated Ag-CSRG. The sandwiched system was then exposed to UV light in an enclosed UV chamber (Novascan PSD-UV, Novascan Technologies Inc., Ames, IA, USA) for 30 min. When the epoxy was cured, the patterned silver was stripped from the Ag-CSRG by a simple peel-off. The stripped substrate, consisting of the smooth Ag nanogratings, was subjected to a final rinsing with 10% ethanol and DI water to dissolve and remove any remaining gDR1 from the metal surface. The cleaned substrate was then air-dried and stored in a microscope glass slide holder for further use.

### 2.4. Raman Measurements

A Horiba/Jobin-Yvon Raman spectrometer (Model: LabRAM) with a 632.8 nm HeNe laser (17 mW), 1800 1/mm grating and an Olympus BX-41 microscope system were used. The collection of spectra was performed in the backscattered mode under the following conditions: ×100 microscope objective, 500 μm pinhole, 500 μm slit width, laser filter 10×, for a sampling time of 10 seconds with 10 repeats. All Raman spectra were background corrected through polynomial subtraction, and the noise was reduced with a Savitsky–Golay filter.

### 2.5. Analyte Sample Preparation

R6G was dissolved in methanol at a stock concentration of 0.1 M and diluted in methanol to generate solutions in the range of 1 µM–1 mM. Melamine was dissolved in Millipore® water to a stock concentration of 1 mg mL^−1^ (1000 ppm) and diluted in water to generate solutions in the range of 1–100 ppm.

### 2.6. Contact Angle Measurements

Contact angle measurements were performed using an OCA 15EC digital goniometer (DataPhysics, Charlotte, NC, USA). Droplets (volume of 2.56 ± 0.13 μL, n = 5) of Nanopure water were dispensed onto a 500 nm Ag TS-CSRG at standard conditions using an electronically controlled syringe. The resulting contact angle was calculated using the SCA 20 software module (DataPhysics, Charlotte, NC, USA) with a Young-Laplace fitting feature for the sessile drop method.

### 2.7. Enhancement Factor Calculations

SERS EF for R6G molecule absorbed on Ag-CSRG was calculated using the following equation [46]:

*EF* = (*I_SERS_*/*N_SERS_*)/(*I_Bulk_*/*N_Bulk_*), (2)

*I_SERS_* and *I_Bulk_* are the intensities of the 1358 cm^−1^ peak with SERS and normal Raman (flat Ag surface), respectively. *N_Bulk_* is the number of molecules illuminated in bulk, giving a normal Raman signal, and *N_SERS_* is the number of molecules illuminated on the nanostructured metallic substrate, giving the SERS signal. The peak at 1358 cm^−1^ represents intensity at a characteristic band wave number for R6G absorbed on an Ag-CSRG and a flat Ag substrate.

### 2.8. Finite-Difference Time-Domain (FDTD) Simulations

Three-dimensional FDTD was used to simulate the distribution of the near-field electromagnetic field on the surface of the TS-CSRG using Lumerical FDTD Solutions software. Simulations under S and P polarizations were recorded and added to emulate the plasmonic response under a quasi-unpolarized broadband excitation light source. Symmetric and antisymmetric boundary conditions were set for the x- and y-directions, respectively, and a perfectly matched layer (PML) in the z-direction. The dielectric permittivity used in the simulations for the UV-curable epoxy and silver were obtained from the manufacturer and the literature, respectively [47]. The topography of a CSRG was modeled according to the following function:

*f*(*x*,*y*)*=A/*2{|*sin*[(4*π*/*p*)*x*]| − |*cos*[(4*π*/*p*)*x*]| − *|sin*[(8*π*/*p*)*x*]*| + |*(*|sin*[(4*π*/*p*)*x*]| −

|cos[(4π/p)x]|)| + |sin[(4π/p)y]| − |cos[(4π/p)y]| − |sin[(8π/p)y]| + (3)

|(|*sin*[*(*4*π*/*p*)*y*]| − |*cos*[(4*π*/*p*)*y*]*|*)*|-cos*[(8*π*/*p*)*x*] − *cos*[(8*π*/*p*)*y*]}

where *A* and *p* correspond to the amplitude and period of the structure, respectively, in accordance with the AFM characterization. A uniform mesh size of 3 nm was used for the envelope of the nanostructure, comprising the UV-curable epoxy, the silver film, and the dielectric medium in all the directional axes. A time-averaged electric field intensity distribution, normalized with respect to the incident plane wave |*E*/*E*_0_|^2^, was calculated for the Ag-CSRG. A frequency-domain field profile is placed at the *xy* plane of the CSRG. To match experimental conditions, |*E*/*E*_0_|^2^ was recorded at 632.8 nm, corresponding to the excitation wavelength of the Raman apparatus.

### 2.9. Scanning Electron Microscopy

High-magnification image acquisition of the surface of TS-CSRGs was achieved using a high vacuum scanning electron microscope (SEM) Quanta FEG 150 ESEM (Field Electron and Ion Company, Hillsboro, OR, USA) with BF/DF STEM detector, at 10 kV. Images of TS-CSRG of 450, 500, 550 and 600 nm were acquired at magnifications of 16,000×, 20,000× and 25,000× (images of all TS-CSRGs are provided in the Supplementary Information).

## 3. Results and Discussion

**Figure 1:**
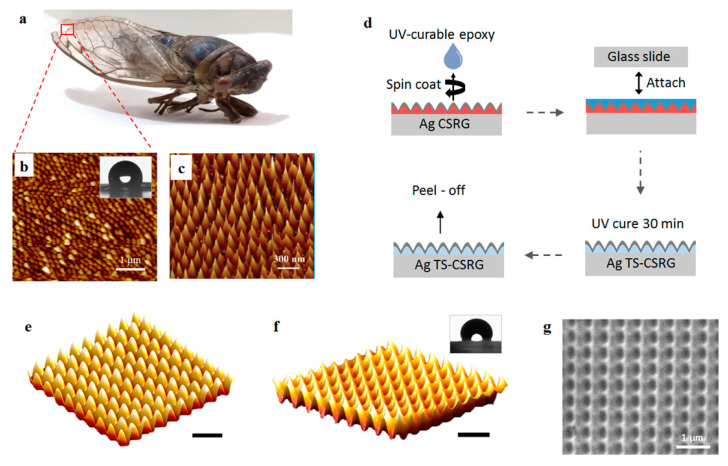
(**a**) Picture of a cicada *Neotibicen canicularis*. (**b**) atomic force microscope (AFM) image of the external surface from a piece of the wing of the cicada; inset: wetting state of a water droplet on a cicada *Neotibicen canicularis.* (**c**) AFM image of the external surface of the wing of a cicada *Cryptotympana atrata fabricius*. Reprinted with permission [48]. Copyright 2017, The Royal Society of Chemistry. (**d**) Schematic representation of the fabrication procedure for creating template-stripped Ag template-stripped crossed surface relief grating (TS-CSRG). AFM scan of a 5 µm × 5 µm area of a 500-nm-pitch (**e**) Ag CSRG, and (**f**) Ag TS-CSRG; inset: wetting state of a water droplet on an Ag TS-CSRG; scale bars correspond to 1 µm. (**g**) SEM image of a 500 nm-pitch Ag TS-CSRG.

A piece of the external façade of the wing of a natural cicada *Neotibicen canicularis* was scanned using atomic force microscopy (AFM). Figure 1a shows a digital picture of the cicada, and Figure 1b shows the AFM scan image of the external surface of a distal portion of the wing. The inset shows an image of a droplet atop the wing of the cicada, acquired during contact angle measurements (more details can be found in the Supplementary Information). Even when the AR nature of the nanostructured cicada wings is dictated by evolutionary survival strategies, the topography between different species of cicadas may vary. Figure 1c shows an AFM scan of the external wing topography of a wing of cicada *Cryptotympana atrata fabricius* reported previously [48].

As the nanostructure pattern in the cicada wing is nearly complementary to a CSRG, it can be reproduced via template-stripping to create the bioinspired SERS-active substrate. The fabrication procedure of the TS-CSRG is schematically shown in Figure 1d. First, surface relief gratings (SRGs) were fabricated by dissolving photoactive gDR1 in dichloromethane, followed by a spin-coating step on a pre-cleaned microscopic glass to achieve a uniform thin film of ~200 nm. Using a laser, gratings with the desired pitch were written on the gDR1-coated substrate by direct holographic exposure to an interference pattern as reported elsewhere [19]. CSRGs were achieved by the in-plane, orthogonal superposition of two sequentially inscribed SRG, as detailed in the Experimental section. Nanometer-level precision in the periodicity of the CSRG is achieved by controlling the fabrication parameters, including laser power, exposure time, and angular position of the sample. The precise control in the periodicity enables the creation of a tailored pitch in ~6 min. A 50-nm thick layer of Ag was deposited on the CSRG to provide the metallic interface for the SPR excitation. Figure 1e shows an AFM scan of a 500-nm-pitch Ag-coated CSRG. The final TS-CSRGs were achieved by selective lift-off of the Ag layer from the CSRG. UV-curable epoxy was spin-coated on the Ag CSRG and then pressed against a pre-cleaned microscopic glass slide and placed in a UV curing chamber for 30 min to allow the epoxy to solidify. The Ag nanostructures were then peeled off and cleaned with ethanol to dissolve any remnant gDR1. This method provided a large-area and smooth Ag TS-CSRG with a complementary pattern of the CSRG, as shown in the AFM scan in Figure 1f. Images on the template stripping process and the resulting nanostructures are shown in Figure S2 in the Supplemental Materials. The inset shows an image of a droplet atop the TS-CSRG, acquired during contact angle measurements. Additional information on the wetting state of the cicada wing and the fabricated nanostructures can be found in the Supplementary Materials, along with images of droplets atop the surfaces taken during the CA measurements (Supplementary Materials Figure S1). Figure 1g shows an SEM image of a 500-nm-pitch Ag TS-CSRG, where the valleys and peaks of the nanostructures are recognizable, analogous to the topology revealed by the AFM scan. Additional SEM images of the Ag TS-CSRGs are also shown in Figure S3 in the Supplementary Materials. Notably, the fabricated TS-CSRGs have a remarkable resemblance to the nanostructures on the wing of cicada *Cryptotympana atrata fabricius*. The nanostructures have a total sensing area of approximately ~1 cm^2^, allowing for a large-area approach for target analyte detection, in contrast to established hot spot methods.

The nanostructures on the wings of the cicadas not only provide them antireflection but also self-cleaning and antibacterial properties that arise from the hydrophobicity of the surface [9]. Compared to a flat silver substrate, the TS-CSRG allows for a metal-dielectric interface with nanoscopic features that significantly alter the wettability of the surface. Typically, the contact angles (CA) of non-wetting surfaces range between 90° and 180°, whether a perfect wetting surface is 0°. An ideal flat silver surface is perfectly wetting; although the CA can vary depending on the cleanness of the surface, it is significantly low compared to values for non-wetting surfaces [49]. The nanostructured features of Ag TS-CSRGs induce a Wenzel state, where the surface exhibits the apparent CA of a non-wetting surface [50], similar to the self-cleaning hydrophobic surface of cicada wings. We investigated the wettability of the TS-CSRG and cicada wing by measuring the static CA using microscopic droplets of DI water. The insets in Figure 1b,f show, respectively, images of droplets on the external surface of a piece of a cicada *Neotibicen canicularis* wing and atop a pristine TS-CSRG taken with the automatic CA measurement system. From the images, it is qualitatively evident the hydrophobicity exhibited by both surfaces. Quantitatively, the measured CA from the cicada wing and the TS-CSRG were, respectively 115° ± 2.075° and 119° ± 3.4222° (n = 5).

Electromagnetic enhancement is critical for SERS-based detection. In a backscattering approach, a surface-confined enhancement assisted by SPR excitations allows for the enhancement of small molecule Raman signals. However, a SERS substrate needs to be tailored to allow for the excitation depending on the incident laser wavelength. Using Equation (1), for air (n = 1) and assuming normal incidence, the desired pitch of the gratings was calculated to be ~560 nm for a laser excitation wavelength of 632.8 nm. However, any analyte on the surface of the metal will eventually change the dielectric permittivity as perceived by the incident light. Hence, a set of TS-CSRGs with a grating pitch ranging from 450 to 600 nm with a 50-nm pitch increment was fabricated to acknowledge the dielectric change encountered by the incident light on the surface. The nanostructures were strategically designed with those periodicities to minimize the reflection of light for wavelengths shorter than the periodicity, promoting therefore enhanced plasmonic regions for the Raman excitation wavelength of 632.8 nm.

**Figure 2:**
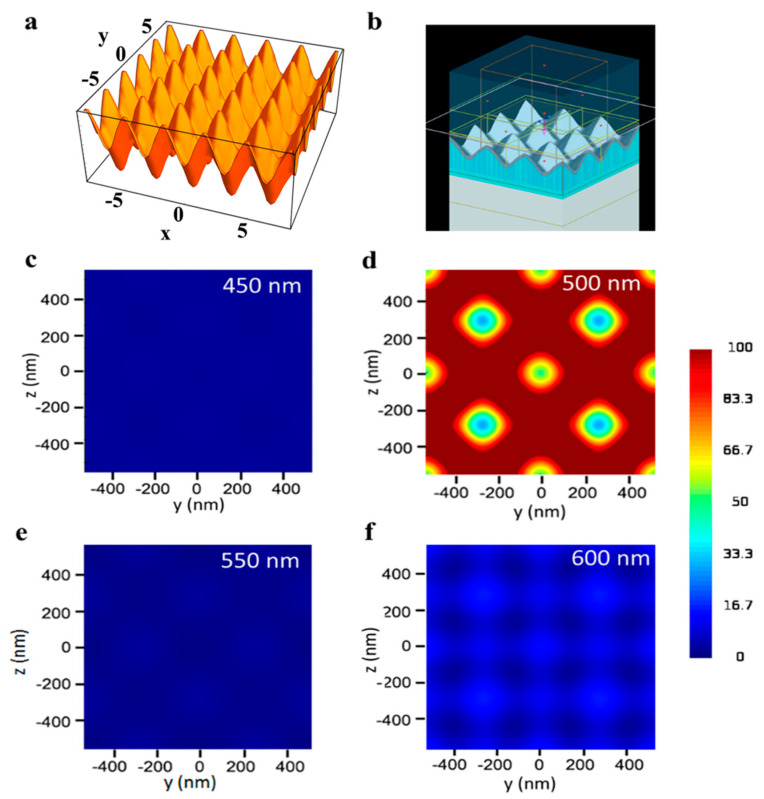
Finite-difference time-domain (FDTD) simulations. (**a**) 3D surface created from Equation (3). (**b**) FDTD simulation model. (**c**–**f**) Electric field distribution along *xy* cross-section for Ag TS-CSRGs with grating pitch spanning from 450 nm to 600 nm, with 50 nm increments.

FDTD simulations were used to demonstrate the polarization-independent electric field enhancement in the vicinity of the TS-CSRG and to confirm the nanostructure pitch leading to the highest EF. Details on the methodology, including the equation utilized to replicate the topography of the nanostructures, are described in the Experimental section. Figure 2a,b shows, respectively, the 3D surface created from Equation (3) and the simulation model used for the FDTD simulations. This equation was utilized to create a model of the TG-CSRGs on Lumerical FDTD solutions software to perform FDTD simulations, which are presented in Figure 2b. The simulations demonstrated the polarization-independent electric field enhancement in the vicinity of the TS-CSRG and confirmed the nanostructures pitch leading to the highest EF. Details on the methodology, including the equation utilized to replicate the topography of the nanostructures, are described in the Experimental section.

Figure 2c–f shows the electric field intensity distribution, |*E*/*E*_0_|^2^, recorded along the *xy* cross-section, for gratings with periodicities spanning from 450 nm to 600 nm for a dielectric with RI of 1.33. All the simulation results were scale-adjusted for intensity values of 0–100. The plasmonic enhancement obtained for all the structures demonstrated to be the same for s- and p-polarized incident light. Figure 2d shows the simulated electric field enhancement of the TS-CSRG of 500-nm pitch, which is at least five times higher than the other periodicities investigated in this work (Figure 2c,e,f). Additionally, it can be observed that the highest electric field enhancement occurs at the crests of the nanostructures. The strength of the electric field decreases in a quasi-radial pattern towards the center of the valleys. Although the simulations may indeed vary from real samples on account of alterations in RI or topography, they served to confirm the response for the TS-CSRG periodicities scrutinized in this work.

**Figure 3:**
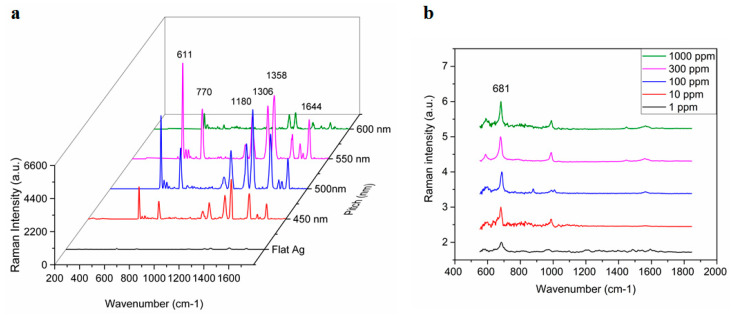
Surface-enhanced Raman scattering (SERS) activity of Ag TS-CSRG. (**a**) Pitch-dependency of SERS spectra for R6G (10^–5^ M) with pitches ranging 450 nm to 600 nm, and for a flat Ag substrate for R6G (10^–2^ M). (**b**) Average SERS spectra of melamine on Ag TS-CSRG for concentrations ranging from 1 ppm to 1000 ppm.

The pitch-dependency of the TS-CSRG was used to tailor the SERS signals response upon the adsorption of Rhodamine 6G (R6G), a Raman reporter molecule with a distinct Raman spectrum. Figure 3a shows the Raman spectra for a flat Ag surface and TS-CSRG with pitches of 450, 500, 550 and 600 nm. Raman peaks at c.a. 610, 770, 1180, 1306, 1360, 1505, 1570, 1595, and 1645 cm^−1^ are characteristic of R6G [51,52]. The reference peak at 1360 cm^−1^, corresponding to aromatic C-C stretching [53], is commonly used as a reference to track changes on the surface of the substrate, and it can be clearly observed in all CSRGs. However, the peak is more prominent in the 500-nm-pitch grating, concurring with the simulation results shown in Figure 2c–f. The SERS enhancement factor (EF), which correlates to the evaluation of signal intensities observed from SERS-active and passive substrates (i.e., flat Ag substrate), was calculated. The Raman vibration of R6G at 1358 cm^−1^ was used for the EF calculations, and the corresponding intensity for R6G (10^−2^ M) on a flat Ag substrate was calculated to be 70 arbitrary units (a.u.). The intensities at 1358 cm^−1^ were recorded for each of the substrates with different pitches using R6G (10^–5^ M). The EF values were calculated using Equation (2) for TS-CSRG with pitches 450, 500, 550 and 600 nm were 3.8 × 10^4^, 7.6 × 10^4^, 6.1 × 10^4^ and 1.6 × 10^4^, respectively. The TS-CSRG with a pitch of 500 nm exhibited the highest EF—a value that may serve as a guideline for SERS detection applications and further investigations, with magnitude comparable to reported values for grating-based SERS substrates [54] and commercial SERS substrates [30].

The TS-CSRG was further evaluated in a real-world detection scenario for the detection of melamine. Melamine is a toxic, nitrogen-rich (66% by mass) chemical used in the plastics industry for the production of compounds for molding, coating, adhesives, and glues. Due to its high nitrogen content, it is illegally added to foodstuffs such as pet food, milk, infant formula to inflate the apparent protein content of the food [55]. Melamine contamination is virtually undetectable by standardized tests as they rely on the amount of nitrogen in test samples as a proxy for the amount of protein. Illegal contamination of dairy products led to severe health problems, resulting in renal failure and even death, with the hospitalization of over 50,000 infants in some cases [56]. The World Health Organization dictates 2.5 ppm (2.0 × 10^−5^ M) as the maximum residue limit for melamine in milk and 1 ppm (7.9 × 10^−6^ M) as the maximum residue limit in infant formula [57]. Detection of melamine usually involves laborious, expensive, and time-consuming methods such as HPLC and LC-MS. In spite of the low detection limit of those methods, they involve immovable heavy equipment and sample preprocessing that make them impractical for point-of-use testing. Melamine has a strong characteristic Raman peak associated with the in-plane deformation of the triazine ring peak (around 676–690 cm^−1^), depending on reaction conditions [58]. This provides an opportunity to allow for the detection of melamine using the Ag TS-CSRG SERS substrate. Melamine in water with concentrations ranging 1 ppm–1000 ppm were drop-casted on the Ag TS-CSRG, followed by SERS spectra acquisition. Figure 3b shows the normalized acquired spectra for melamine for the different concentrations. The characteristic Raman peak associated with the in-plane deformation of the triazine ring peak is distinguishable up to 1 ppm, a concentration that is in line with the WHO regulations for melamine in food products. These results demonstrate that the Ag TS-CSRG presented here can be used as an inexpensive, yet effective SERS sensor with a topology that can be customized to transmit or reflect specific light wavelengths, similarly to actual nanostructures in cicada wings, to enable signals tailored to employ and acquire specific wavelengths for sensing.

## 4. Conclusions

In conclusion, this work presents a new Ag TS-CSRG as polarization-independent SERS active substrate, inspired by the tapered nanopillars found in cicada wings. The fabrication of the substrate is cost-effective and achieved in a cleanroom-free environment via holographic exposure followed by a template-stripping step using a UV-curable resin. Inspired by the AR properties of the cicada wings, the nanostructures are strategically designed to minimize the reflection of light for wavelengths smaller than their periodicity, promoting enhanced plasmonic regions for the Raman excitation wavelength at 632.8 nm. AFM scans reveal that the TS-CSRGs possess a remarkable resemblance to the nanostructures in the wings of cicada *Cryptotympana atrata fabricius* and are equally hydrophobic, providing them with potential self-cleaning and bactericidal properties. The nanostructures enable a field enhancement that allows for the sensitive and reproducible SERS detection of R6G. Simulations and experimental investigation of the SPR-assisted electromagnetic enhancement are performed via FDTD and detection of SERS-active dye R6G, respectively, to validate the nanofabrication parameters. More important, the TS-CSRG enables the label-free, sensitive detection of melamine at concentrations compatible with the maximum residue limits allowed by the WHO in food. The fabrication methodology of TS-CSRG allows for the generation of nanostructures with customized periodicities that can be tailored for specific applications. Therefore, the new bio-inspired functional, SERS-active TS-CSRG introduced here holds great promise as large-area, label-free SERS-active substrates for medical and biochemical sensing applications.

## Data Availability

Not applicable.

## Supplementary Materials

The following are available online at https://www.mdpi.com/1424-8220/21/5/1699/s1, Figure S1: Images taken during contact angle measurements of a DI water droplet atop the surface of a piece of wing from a cicada Neotibicen canicularis and of an Ag TS-CSRG, Figure S2: CSRGs template stripping process, Figure S3: SEM images of the Ag TS-CSRGs with pitches of 450 nm, 500 nm, 550 nm and 600 nm.
