# Supplementary material for: Cicada Wing Inspired Template-Stripped SERS Active 3D Metallic Nanostructures for the Detection of Toxic Substances
Source: Sensors (Basel). 2021 Mar 2;21(5):1699. doi: 10.3390/s21051699 (PMC7957863; doi:10.3390/s21051699)
Supplement: Supplementary file 1 [file sensors-21-01699-s001.pdf]

sensors

MDPI

Supplementary Materials

Cicada Wing Inspired Template-Stripped SERS Active 3D Metallic Nanostructures for the Detection of Toxic Substances

Srijit Nair [1,†], Juan Gomez-Cruz [1,2,†], Gabriel Ascanio [2], Aristides Docoslis [1], Ribal Georges Sabat [3] and Carlos Escobedo [1,*]

[1] Department of Chemical Engineering, Queen's University, Kingston, ON K7L 3N6, Canada; srijit.nair@queensu.ca(S.N.); j.gomezcruz@queensu.ca(J.G.-C.); docoslis@queensu.ca(A.D.)
[2] Instituto de Ciencias Aplicadas y Desarrollo Tecnológico (ICAT), Universidad Nacional Autónoma de México (UNAM), Cto. Exterior S/N, C.U., Coyoacán, Ciudad de México 04510, Mexico; gabriel.ascanio@icat.unam.mx
[3] Department of Physics and Space Science, Royal Military College of Canada, Kingston, ON K7K 7B4, Canada; sabat@rmc.ca
* Correspondence: ce32@queensu.ca
† These authors contributed equally to this work.
Tel.: +1-613-533-3095; sabat@rmc.ca; Tel.: +1-613-541-6000 ext. 6721

Contact angle measurements

The wetting state of surfaces is often used to evaluate bactericidal properties of both bioinspired and natural nanostructured materials [1,2]. In that context, the wetting state of the bioinspired TS-CSRGs and the cicada wings were compared through contact angle (CA) measurements. Figure S1 shows images of the profile of microdroplets on the wings of the cicada *Neotibicen canicularis* and on the TG-CSRG structures, taken during the CA measurements, as described in Section 2.6. The resulting CAs demonstrate similar hydrophobic properties with contact angles of 115° ± 2.075° and 119° ± 3.4222° (n = 5) for the cicada wings and the TS-CSRG, respectively.

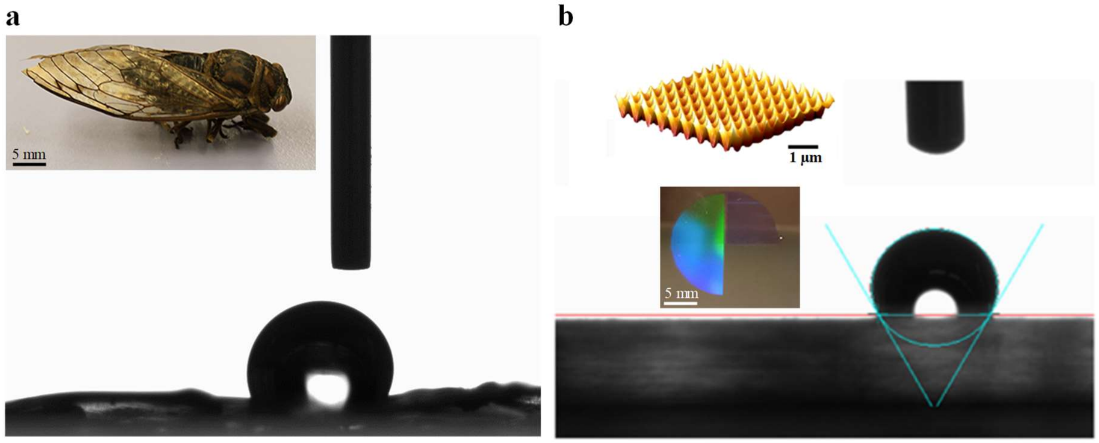

Figure S1. Images taken during contact angle measurements of a DI water droplet atop the surface of (**a**) a piece of wing from a cicada Neotibicen canicularis (inset: cicada used as sample source); and (**b**) an Ag TS-CSRG (inset: TS-CSRG, left; AFM scan, right).

Preparation and inspection of the TS-CSRG SERS substrates

The template stripping process to fabricate the TS-CSRGS is shown in the pictures in Figure S2. Macroscopic and microscopic optical inspection of the nanostructures was performed in order to evaluate the successful preparation of the SERS-active substrates. Figure S2a shows the inscribed and silver coated CSRG (methodology described in Section 2.2). The two orthogonal pitches of the CSRG were verified for accuracy by measuring the first-order diffraction angle from a low-power Helium-Neon laser and by using the grating equation. Subsequently, UV curable epoxy (NOA61, Norland Products Inc., NJ, USA) is spin-coated on top of the silver-coated nanostructures and a glass slide is carefully placed down and pressed uniformly to distribute the glue and remove any air in between. The structure is then exposed to UV light in an enclosed UV chamber (Novascan PSD-UV, Novascan Technologies Inc., IA, USA) for 30 min (Figure S2b). Once cured, the glass slide is meticulously removed to transfer the silver pattern. The excess of gDR1 is removed by rinsing with ethanol and DI water. Figure S2c shows Au TS-CSRG of 450, 550 and 600 nm successfully transferred to glass slides, which exhibit light diffraction patterns similar to the original CSRG.

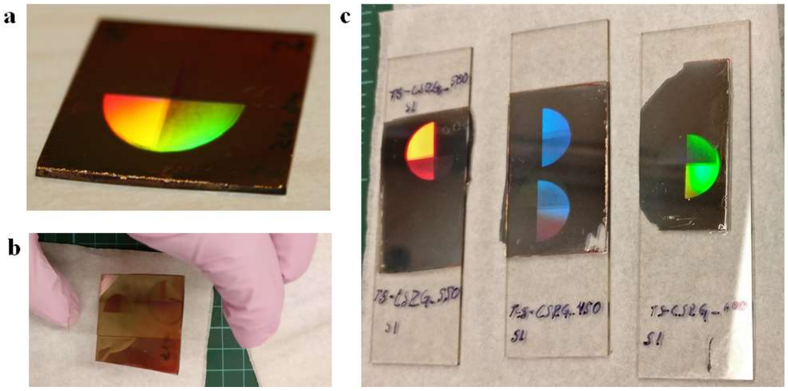

Figure S2. CSRGs template stripping process. (**a**) Silver-coated CSRG structure. (**b**) Glass slide and CSRG structure after spin coating and UV exposure. (**c**) TS-CSRG structures.

Scanning Electron Microscopy (SEM) Characterization

The surfaces of the Ag TS-CSRGs were investigated through the use of a high vacuum Scanning Electron Microscope (SEM) model Quanta FEG 150 ESEM (Field Electron and Ion Company, FEI, Oregon, USA) with BF/DF STEM detector, at 10kV. Figure S3 shows images of TS-CSRG of 450, 500, 550 and 600 nm at magnifications of 16000x, 20000x and 25000x.

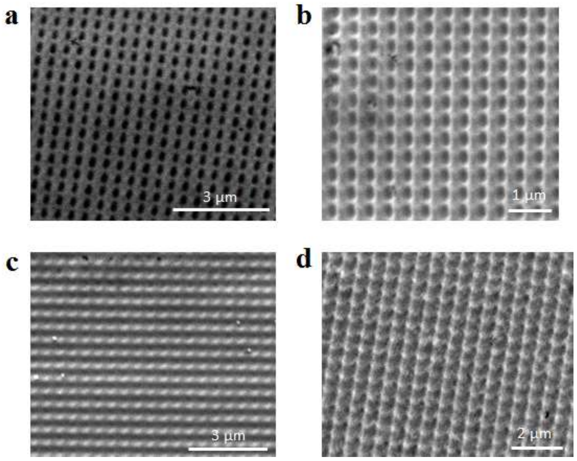

Figure S3. SEM images of the Ag TS-CSRGs with pitches of (**a**) 450 nm, (**b**) 500 nm, (**c**) 550 nm and (**d**) 600 nm.

References

1. lbourne, A.; Crawford, R.J.; Ivanova, E.P. Nano-structured antimicrobial surfaces: From nature to synthetic analogues. *J. Colloid Interface Sci.* 2017, *508*, 603–616.
2. Hasan, J.; Webb, H.K.; Truong, V.K.; Pogodin, S.; Baulin, V.A.; Watson, G.S.; Watson, J.A.; Crawford, R.J.; Ivanova, E.P. Selective bactericidal activity of nanopatterned superhydrophobic cicada Psaltoda claripennis wing surfaces. *Appl. Microbiol. Biotechnol.* **2013**, *97*, 9257–9262, doi:10.1007/s00253-012-4628-5.
